# Spatial-temporal distribution characteristics of haemorrhagic fever with renal syndrome (HFRS) in 31 provinces and municipalities of P.R. China and multi-factorial prediction of incidence

**DOI:** 10.1371/journal.pntd.0014619

**Published:** 2026-09-11

**Authors:** Huan Huang, Xin Zhao, Jiaojiao Qian, Hengduan Zhang, Fanjin Meng, Qiyuan Kuai, Weibo Ma, Yifan Wu, Huajing Zhu, Weilong Tan

**Affiliations:** 1 Department of Epidemiology, School of Public Health, Nanjing Medical University, Nanjing, China; 2 Nanjing Bioengineering (Gene) Technology Centre for Medicines, Nanjing, China; 3 Jinling Hospital, Nanjing, Jiangsu, China; 4 State Key Laboratory of Pathogen and Biosecurity, Beijing, China; Australian Red Cross Lifeblood, AUSTRALIA

## Abstract

**Objective:**

To integrate multi-source spatiotemporal data and construct an HFRS incidence prediction model using XGBoost with SHAP, exploring factors associated with HFRS incidence in China.

**Methods:**

Collected HFRS case data from 31 provinces in China (2005-2020) and multi-source data on climate, land use, and population. Used MaxEnt to analyze rodent distribution and key environmental variables. Constructed an XGBoost model integrating climate, land use, host, and socio-economic features, with SHAP values assessing feature contributions. Dataset divided into training (70%), validation (15%), and test (15%) sets, with higher weights for high-incidence provinces.

**Results:**

The reported HFRS cases in mainland China decreased from 2005 to 2020 but showed seasonal peaks. Cases were mainly among adults aged 20-60 years. The distribution of virus-carrying rodents was mainly affected by minimum temperature of the coldest month and elevation. After removing temporal features, land-use types (forest, cropland, shrub) showed greater relative importance than climate variables.

**Conclusion:**

Forest, shrub, and cropland may affect disease transmission via host distribution and food availability. Climate factors act indirectly on HFRS risk. Temporal features remain critical for incidence prediction, while land use and rodent habitats are key exogenous drivers. These findings support targeted public health strategies.

## Author summary

Hemorrhagic fever with renal syndrome (HFRS) is a serious rodent-borne infectious disease that remains an important public health concern in China. Although the number of reported HFRS cases has generally declined, the disease continues to show clear seasonal and geographical differences, making it difficult to identify areas and periods of increased risk. In this study, we combined HFRS surveillance data from 31 provincial-level regions in mainland China between 2005 and 2020 with information on climate, land use, population, and the distribution of virus-carrying rodents. We found that HFRS cases were concentrated mainly among adults aged 20-60 years and showed distinct seasonal peaks. We also identified important differences in the distribution of the two major rodent groups associated with HFRS. Our prediction analysis showed that recent HFRS case patterns were the strongest predictors of future incidence. When previous case information was excluded, land-use characteristics, particularly forest and cropland, became the most important factors, while climate factors appeared to influence HFRS risk partly by shaping rodent habitats. These findings highlight the importance of combining disease surveillance with environmental and rodent monitoring to improve early warning and support more targeted HFRS prevention and control in China.

## Introduction

Hemorrhagic Fever with Renal Syndrome (HFRS), also known as epidemic hemorrhagic fever, is one of the most burdensome rodent-borne diseases globally. Its pathogens, Hantavirus (HVs) or Seoul virus (SEOV), have established stable natural foci across the Eurasian continent [1–3]. These viruses are transmitted to humans through exposure to contaminated rodents or infectious aerosols generated by these animals [4]. Upon infection, patients typically present with symptoms such as fever, headache, myalgia, renal damage, and hemorrhagic manifestations [5]. Among the approximately 60,000–100,000 infection cases reported globally each year, over 90% occur in China [6]. In recent years, the overall number of reported HFRS cases in mainland China has been on a downward trend [7]. This highly aggregated epidemic pattern is closely related to complex ecological environments. The breeding cycles of host rodents (such as *Rattus norvegicus* and *Apodemus agrarius*) are driven by climatic fluctuations, while land-use changes influence the probability of host-human contact by altering habitat structures [8,9]. More importantly, China’s vast geographical differences and diverse climate types make the elimination of HFRS extremely challenging. In the high-incidence areas of northern China [7,10,11], a strengthening of the “bimodal” seasonal pattern has been observed over the past decade, creating an urgent need for precise early warning systems.

The known risk factors for HFRS incidence include climate, host population dynamics, and viral epidemiology, with climate being widely recognized as an important factor [12,13]. It primarily affects the prevalence of the virus and the risk of human infection by influencing rodent population dynamics. For example, temperature, humidity, and precipitation affect crop yields, which are a food source for rodents [13,14]. Moreover, the dynamics of rodent population density and Hantavirus infection prevalence are related to contact rates. The virus may become extinct below a certain host density [12,15,16]. Under the context of global warming, the periodic dynamics of rodent populations are changing, and new epidemic areas are emerging [17]. There is an urgent need to explore the transmission of HFRS under different climatic conditions [18]. Additionally, human activity patterns, which are influenced by weather conditions and seasonal fluctuations, have a direct impact on human-rodent interactions [19]. Furthermore, there is a positive correlation between population density and HFRS outbreaks. An increase in urbanization rates and gross domestic product (GDP) tends to be associated with higher HFRS incidence rates. Against the backdrop of global climate change, a thorough understanding of the intricate interactions between various variables and the multifaceted determinants affecting HFRS trends in China could be crucial for effectively mitigating the disease.

Thus, we hypothesized that climate conditions, land-use structure, and rodent host distribution jointly and interactively influence the spatiotemporal variation of HFRS incidence in mainland China.

Mathematical models have been widely used for predicting disease transmission [5]. However, existing models often focus on single-factor analyses [20]. For example, time-series models based on climatic factors (such as SARIMA) can capture seasonal patterns but ignore long-term effects brought about by factors such as human activities and land-use changes [21,22]. Landscape composition is an important factor in the ecology of the rodent-Hantavirus ecological system [23,24]. Although niche modeling or species distribution modeling can analyze the risks of ecological fragmentation, they struggle to integrate social determinants (such as population movement and urbanization). This “divide and conquer” research paradigm leads to the neglect of key synergistic mechanisms, which may limits the precise allocation of prevention and control resources. XGBoost is a boosting algorithm that combines multiple learning algorithms to achieve competitive predictive performance than any single component learning algorithm, and it has shown satisfactory performance in many fields [25]. Based on multi-source data from 31 provinces and municipalities in China from 2005 to 2020, this study constructed an XGBoost with SHAP feature framework. This framework integrates the static niche model with the dynamic advantages of machine learning to build a provincial prediction model. Spatial interaction terms were generated, and high-incidence areas were precisely modeled through sample reweighting. SHAP was introduced to parse the model’s decision-making path, enabling visualization of important drivers at the provincial scale and guiding targeted intervention measures. The study also explored the impacts of selected factors and their interactions on HFRS incidence.

## Results

### Temporal trends of reported cases and infection status in Mainland China, 2005–2020

**Fig 1:**
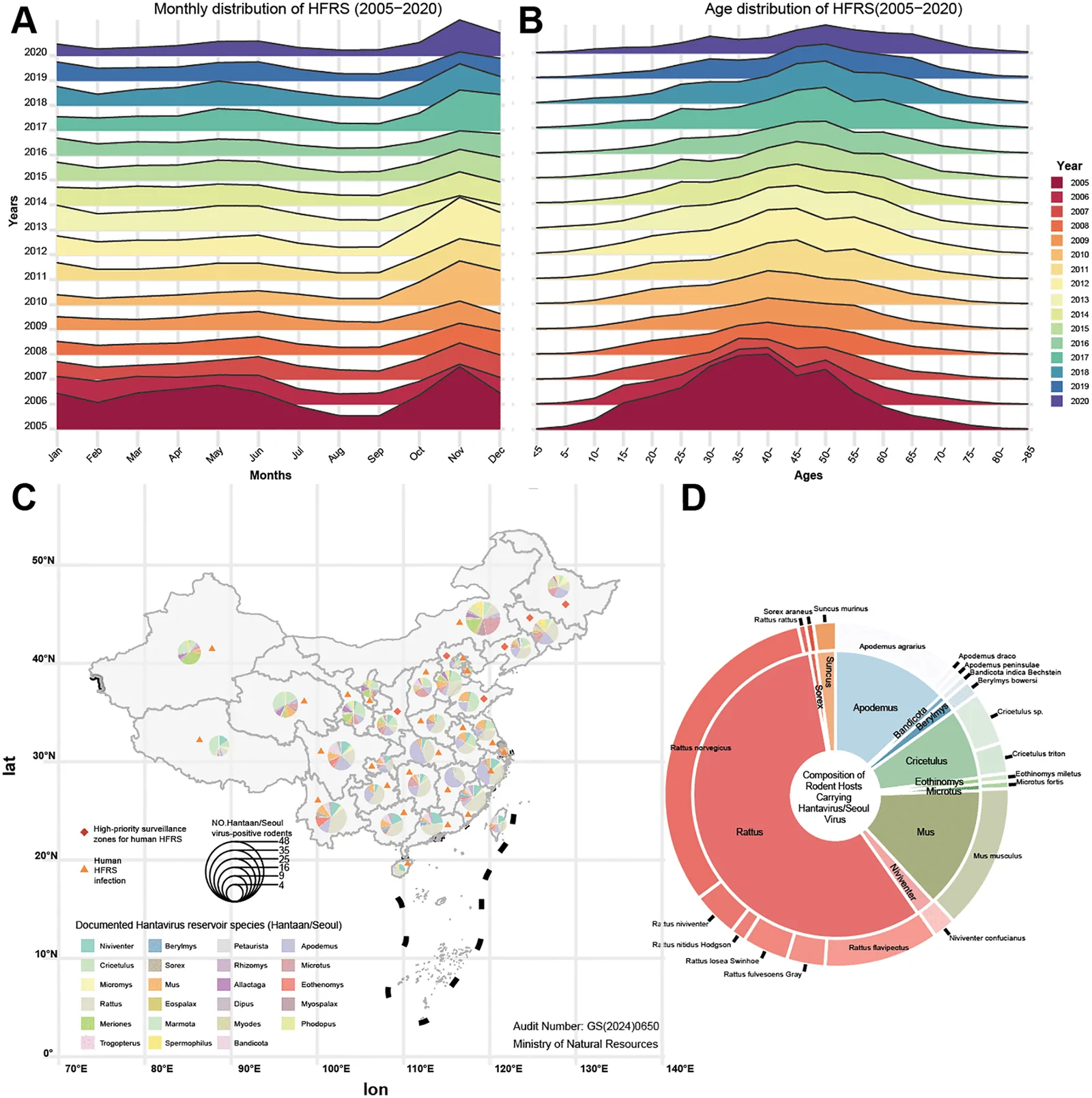
Density plots of HFRS age and seasonal distribution, and distribution maps of rodents documented in the literature. Figure size: 240.42 mm × 132 mm, 300 dpi. **A)** Density plot of monthly distribution of reported HFRS cases from 2005 to 2020; **B)** Density plot of age distribution of reported HFRS cases from 2005 to 2020; **C)** Distribution map of rodents across the country; **D)** Sunburst diagram of rodent species carrying HV/SEOV. The base map uses province and county-level administrative boundaries from the geoBoundaries Global Database (https://www.geoboundaries.org/countryDownloads.html); license: CC BY 4.0 (https://www.geoboundaries.org/#tabs1-js).

From 2005 to 2020, a total of 182,865 cases were reported in mainland China, with the majority of cases concentrated in Heilongjiang Province, Liaoning Province, Shandong Province, Shaanxi Province, Jilin Province, and Hebei Province (Fig 1C). Overall, the reported cases of HFRS in mainland China showed a downward trend but with distinct seasonal peaks (Fig 1A). Notably, a significant peak in reported cases was observed in November and December during the period from 2012 to 2020 (Fig 1A). In terms of the age distribution of reported cases, the incidence was primarily concentrated among adults aged 20–60 years, with the age group of 30-50 years typically experiencing the highest incidence (Fig 1B). Although the overall reported cases decreased, the proportion of reported cases among individuals aged 60 years and above showed a relative increasing trend (Fig 1B).

### Distribution and pathogen carriage of rodents in China

In this study, we included over 7,000 rodent records, of which 1,500 were obtained from NCBI and more than 6,000 were derived from Chinese literature. Among the top 20 rodent genera (accounting for 90.8% of all records), the genus *Rattus* had the highest proportion at 28.2%, followed by the genus *Apodemus* at 15.9% (Fig 1C, S1 Table). At the provincial level, Yunnan Province had the highest proportion, accounting for 13.9%. Previous studies have indicated that *Apodemus* and *Rattus* are important hosts for Hantavirus and Seoul virus. Spatial autocorrelation analysis of these two genera revealed a significant positive spatial correlation in their distributions, indicating strong spatial clustering of both genera in China. However, the degree of clustering was slightly lower for *Apodemus* (Moran’s I = 0.214, p < 0.05) than for *Rattus* (Moran’s I = 0.253, p < 0.01).

### Distribution and ecological niche prediction of rodents carrying HV/SEOV carriers

Among over 7,000 rodent distribution records, 1,640 records of HV/SEOV-positive rodents were identified, mainly concentrated in Zhejiang, Shandong, Hunan, and Hebei provinces (Fig 1C). These rodents belonged to 11 genera and 20 species, with *Rattus norvegicus* accounting for the highest proportion (31.7%), followed by *Mus musculus* (13.7%), *Apodemus agrarius*, and *Rattus flavipectus* (each approximately 11%) (Fig 1D).

**Fig 2:**
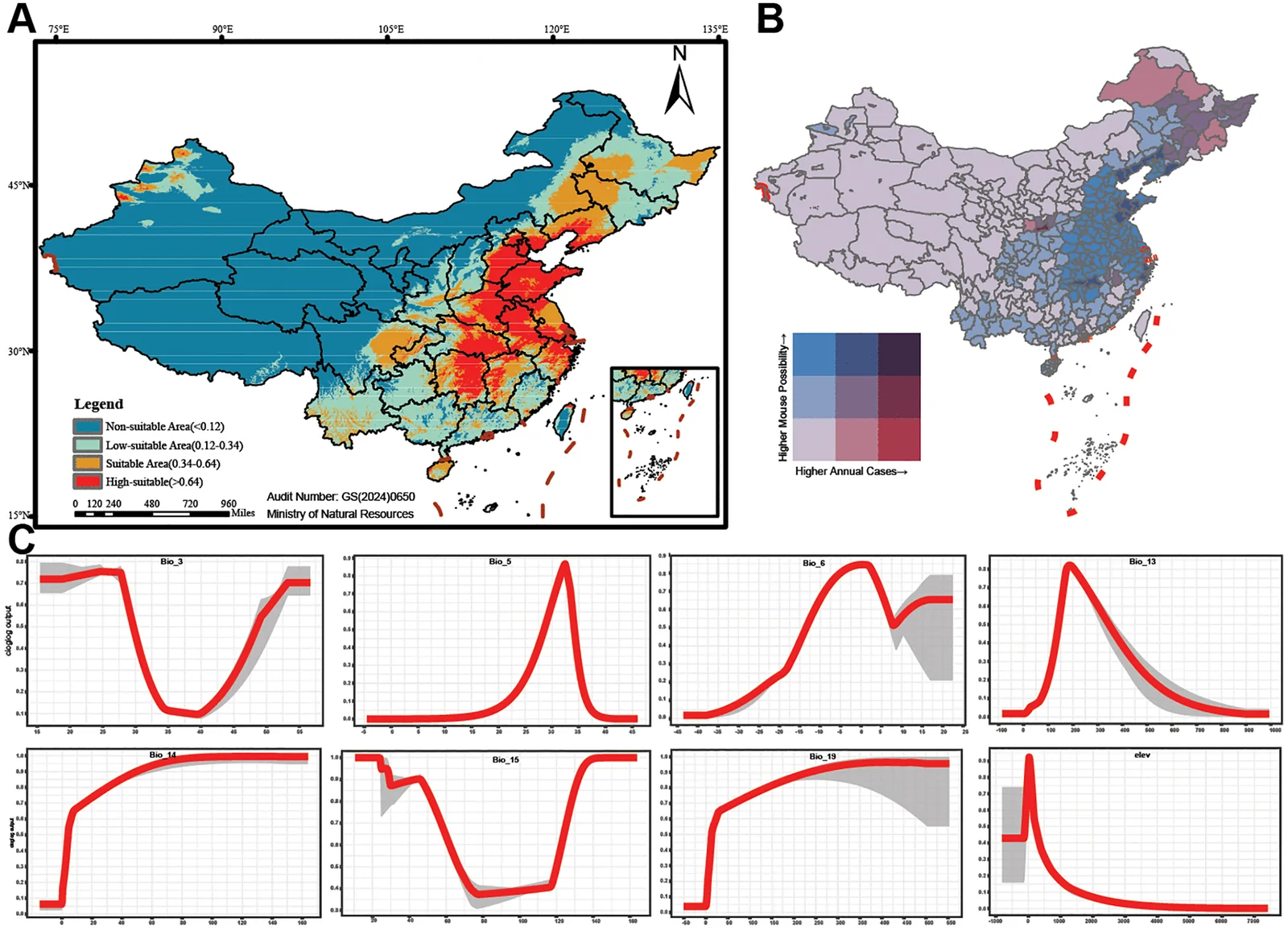
Distribution and ecological niche prediction of HV/SEOV carriers and response curves. Figure size: 137.56 mm × 132 mm, 300 dpi. **A)** Ecological niche prediction of rodents HVs/SEOV carrier; **B)** Bivariate map of HFRS incidence mean and rodent carrying HV/SEOV suitability from 2005 to 2020; **C)** Response curves from MaxEnt. *Bio_13 = Precipitation of Wettest Month; Bio_14 = Precipitation of Driest Month; Bio_15 = Precipitation Seasonality (Coefficient of Variation); Bio_19 = Precipitation of Coldest Quarter; Bio_3 = Isothermality (BIO2/BIO7) (×100); Bio_5 = Max Temperature of Warmest Month; Bio_6 = Min Temperature of Coldest Month; The base map uses province and county-level administrative boundaries from the geoBoundaries Global Database (https://www.geoboundaries.org/countryDownloads.html); license: CC BY 4.0 (https://www.geoboundaries.org/#tabs1-js).

The MaxEnt model achieved an average test Area Under the Curve (AUC) of 0.896 (S1 Fig). Key influential variables were Bio_6 (Minimum Temperature of the Coldest month, 8.32%), Bio_15 (Precipitation Seasonality, 11.073%), Bio_13 (Precipitation of Wettest Month, 10.79%), and ELEV (Elevation, 61.20%) (S2 Table). Jackknife analysis indicated that elevation appeared to be a strong predictor, while Bio_15 has the weakest predictive ability (S2 Fig). Response curve analysis suggested a negative correlation between elevation and carrying rodent suitability; suitability decreased with increasing elevation (S3 Fig). Virus carriage likelihood increased with Bio_14 (Precipitation of Driest Month) and Bio_19 (Precipitation of Coldest Quarter), and showed unimodal relationships with elevation, Bio_5 (Max Temperature of Warmest Month) and Bio_13 (Fig 2D). High-suitability areas are shown in red (Fig 2B).

### Construction and SHAP interpretation of the National HFRS prediction model based on multi-source data

We developed an XGBoost prediction model integrating multiple features, including climate, land cover, host and social factors using Tweedie regression for zero-inflated monthly case data. Early stopping was applied, with data split into training (20052016), validation (2016-2017) and test sets (2018-2020). The model achieved an *R*^2^ of 0.8006 on the validation set (S3 Table).

**Fig 3:**
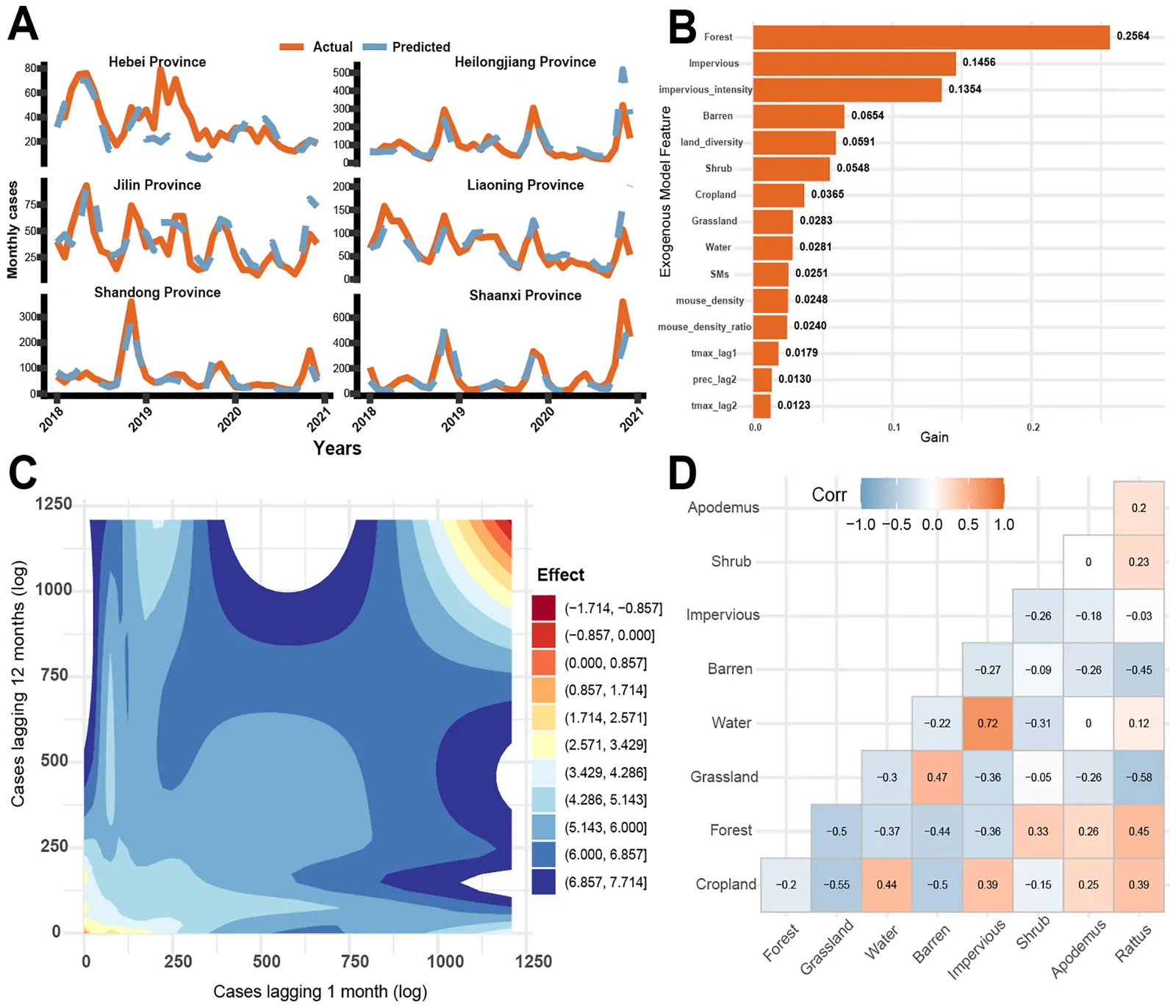
XGBoost prediction model, SHAP interpretation, and analysis of important factors. Figure size: 280.11 mm × 132 mm, 300 dpi. **A)** Differences between the constructed XGBoost prediction model and actual values (2018-2020) (Top 6 provinces); **B)** SHAP interpretation of the XGBoost model without temporal lag variables; **C)** GAM-based interaction surface for the effects of case lag1 and case lag12 on current HFRS incidence (log scale); **D)** Heatmap of correlations between land-use features and *Apodemus* and *Rattus*.

Feature importance analysis showed that temporal lag variables (annual_trend, case_lag1, and case_lag12) contributed most prominently, accounting for more than 80% of total contribution. After removing temporal features to evaluate exogenous drivers, the model achieved an *R*^2^ of 0.7084 (S4 Table). SHAP analysis indicated that land-use types were major contributors, especially Forest (0.2546) and Impervious surface (0.1456) (Figs 3B, S4).

Fig 3C presents the joint effect of case_lag1 and case_lag12 on current HFRS incidence using Generalized Additive Model (GAM). Blue indicates positive effects (higher risk), red indicates negative effects (lower risk). The highest positive effects were observed when both lagged cases were at moderate-to-high levels (600-800 cases). At extremely high values (>1000 cases), the effect became negative, suggesting a nonlinear saturation pattern.

### Impact of land-use types on major hosts

**Fig 4:**
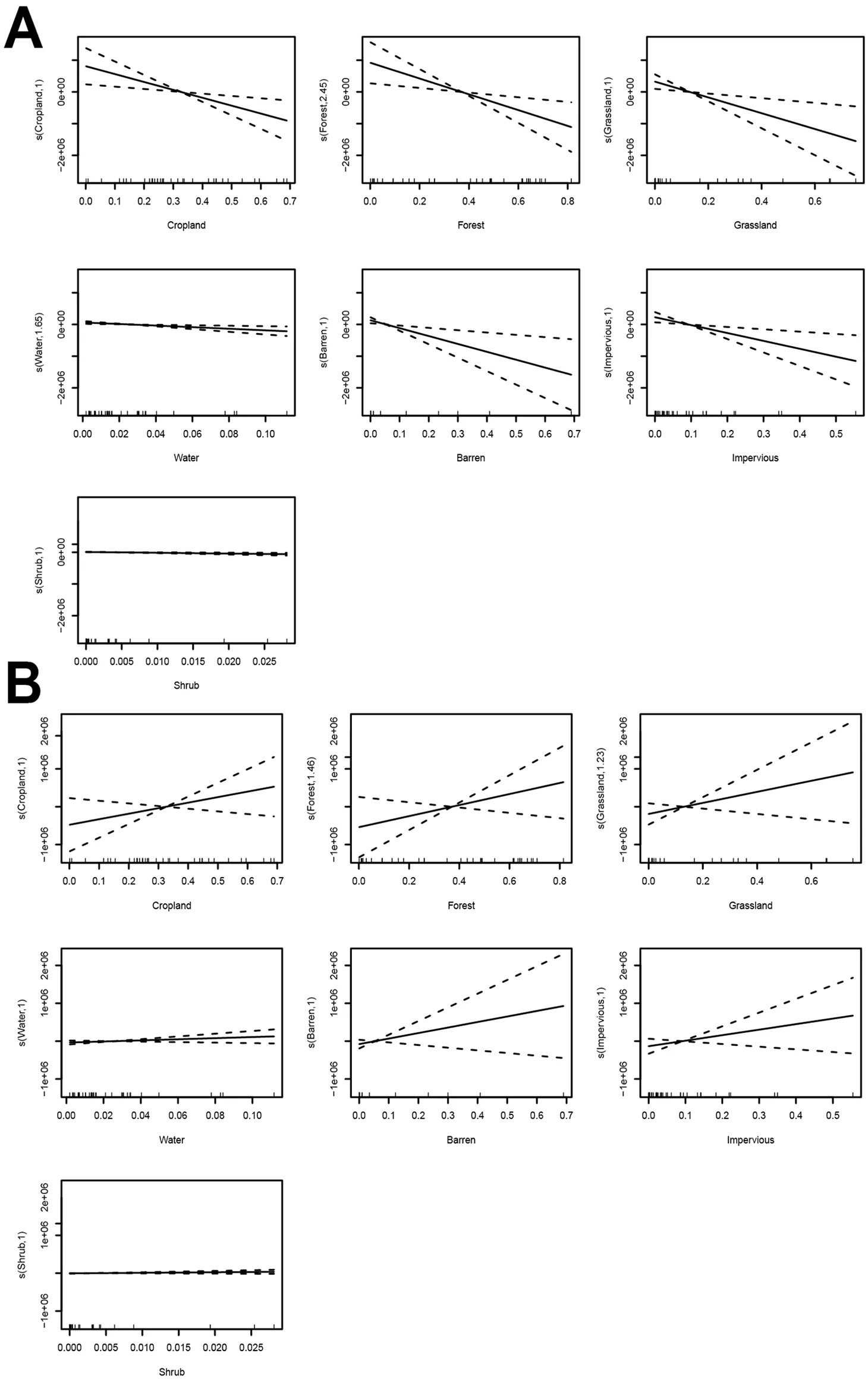
Nonlinear relationships between Apodemus and Rattus and various land-use features. **A)** *Apodemus*; **B)** *Rattus*.Figure size: 178.07 mm × 132 mm, 300 dpi.

Apodemus and Rattus were primary HV/SEOV hosts. Both genera were positively correlated with cropland and forest proportions (Fig 3D). GAM analysis revealed that Apodemus exhibited a low-promotion, high-inhibition relationship with land-use features, while Rattus showed the opposite trend. The effects of water bodies and shrubland were not significant (Fig 4A-4B).

Principal Component Analysis (PCA) analysis showed that among the six provinces, Heilongjiang, Liaoning, and Jilin, were dominated by cropland, Shandong and Hebei provinces by impervious surfaces and water, and Shaanxi by high forest and shrub.

**Fig 5:**
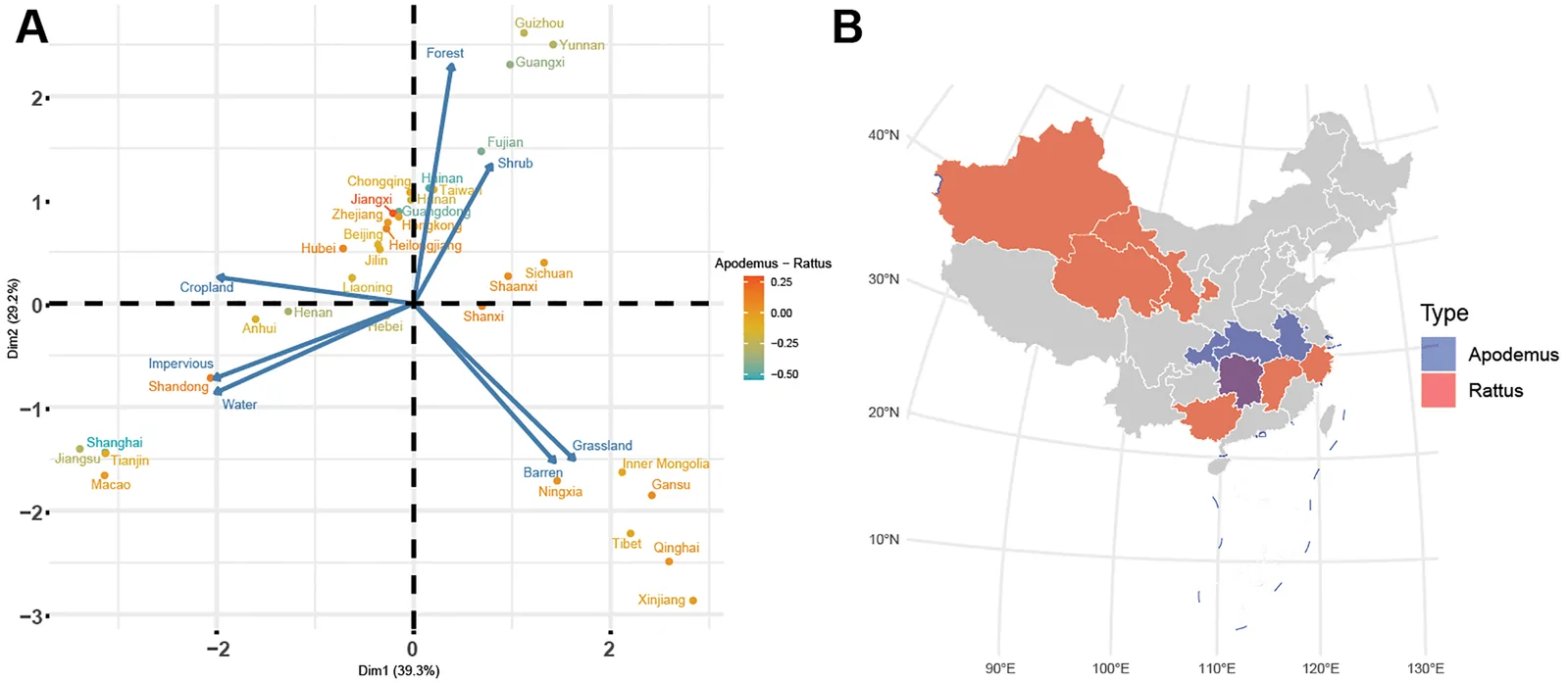
A) PCA Analysis of Land-Use Features and B) LISA Hotspot Analysis of Apodemus and Rattus. The base map uses province and county-level administrative boundaries from the geoBoundaries Global Database (https://www.geoboundaries.org/countryDownloads.html); license: CC BY 4.0 (https://www.geoboundaries.org/#tabs1-js).

Local Indicators of Spatial Association (LISA) analysis identified Hunan as a common hotspot for both genera (Fig 5B). *Rattus* hotspots also included Xinjiang, Shaanxi, Qinghai, Guizhou, Zhejiang, and Shanxi, *Apodemus* hotspots included Anhui, Chongqing, and Hubei.

## Discussion

This nationwide study aimed to investigate determinants of HFRS incidence in mainland of China from 2005 to 2020 and to establish a predictive model for outbreak risk. Based on high-resolution climate and population data, we examined multidimensional factors. In the context of global climate change, our findings help improve understanding of potential mechanisms by which climate, host, and ecological factors may influence HFRS occurrence. We found that lagged case features play a core role in predicting HFRS distribution, followed by land-use features at the case locations, which may indirectly affect disease transmission by modulating rodent distributions. These observations provide a new perspective for understanding the HFRS epidemic drivers.

This study complements and extends previous provincial-scale HFRS research by providing a national-level analysis with multi-factor interactions. Early models such as geographic information systems (GIS) and spatial autocorrelation analysis methods, employing models such as seasonal difference space-time autoregressive integrated moving average (SD-STARIMA), seasonal autoregressive fractionally integrated moving average (SARFIMA), and random forests [2,17,20,26,27] had limitations in integrating temporal and spatial dimensions. In contrast, the XGBoost framework used here better captures complex spatiotemporal patterns in HFRS data. SHAP values indicated that time-series features had relatively high contributions, followed by land-use and climate features.Cropland and Forest showed the strongest impacts among land-use variables and appeared to promote rodent distribution.

By integrating MaxEnt-derived host suitability into the XGBoost framework,we improved the interpretability of HFRS epidemiology. *Apodemus* (carrying Hantaan virus, HTNV) was mainly distributed in wild habitats and associated with higher fatality; *Rattus* (carrying Seoul virus, SEOV) was more urban-associated with spring peaks and lower fatality. Urbanization may increase HFRS risk in Rattus-predominant regions [8,27]. Moreover, urbanization, socioeconomic transformation, and agricultural development have a significant impact on HFRS, especially in the host systems of *Apodemus* and *Rattus* [28]. *Apodemus* showed wider ecological adaptability and was promoted by cropland, forest, and grassland. Although urbanization may favor Rattus distribution [29], our XGBoost model suggested a slight negative interaction between rodent density and impervious surfaces. Improved agricultural practices and public health measures may reduce rodent density and alter HFRS epidemics, highlighting the need for further research. Climate-driven changes in temperature and precipitation may affect rodent survival and abundance [30], which was supported by our MaxEnt results. Overall, monitoring temporal case trends remains important for HFRS prediction and early warning. After excluding temporal lag features, land-use types showed the highest predictive importance, followed by climate variables and host-related factors. No lagged climate terms were included in the exogenous driver model.These findings indicate that landscape structure and elevation play key roles in shaping HFRS risk.

Among the provinces and regions of concern, although Xinjiang, Qinghai, Guizhou, Zhejiang, Shanxi, Anhui, Chongqing, and Hubei are not high-incidence hotspots for reported cases, they are dual high hotspots for rodents (*Apodemus* and *Rattus*), indicating that the risk of HFRS incidence in these areas remains high. Studies have shown that prioritizing the control of peridomestic rodents in spring and forest rodents in late autumn and early winter may provide an effective method for targeting specific Hantaviruses that cause HFRS [27].

Empirical studies have shown that climate factors (including temperature, precipitation, and humidity) have an impact on HFRS [27,28]. Previous studies have continuously emphasized the role of climate variables (especially precipitation and humidity) in HFRS epidemics. With ongoing climate change, the increasing frequency of extreme weather events may further influence HFRS dynamics.

In this study, we examined climate contributions to HFRS incidence and found that the relative importance of climate variables was lower than temporal features and land-use-related features. This observation supports the notion that climate may influence HFRS indirectly, rather than through direct effects, by shaping rodent host distributions and habitats [31–33]. Our MaxEnt model further indicated that the distribution of Rattus carrying HV/SEOV was mainly associated with annual mean temperature and annual precipitation, with precipitation showing a relatively stronger influence. For rodents of the genus *Apodemus*, the main factors are precipitation in the wettest and driest seasons [34]. Taken together, these results support the continued inclusion of temperature and precipitation as important climate‐related risk indicators in HFRS surveillance.

Several limitations should be noted. First, although multiple factors and some interactions were assessed, their causal mechanisms were not fully explored. Second, incomplete virus-carrying records in rodent data may lead to deviations in the MaxEnt model and potentially underestimate host contributions. Third, underreporting may exist due to asymptomatic infections. Fourth, this study was limited to the provincial level, municipal-scale could provide more refined evidence. Despite these limitations, our study provides a foundational framework for predicting future HFRS risks in mainland China.

## Conclusion

This study assessed the spatiotemporal patterns and determinants of HFRS in mainland China from 2005 to 2020 using multi-source data and integrated models. HFRS incidence showed an overall downward trend with obvious seasonal fluctuations and was concentrated among adults aged 20–60 years. Elevation, minimum temperature of the coldest month, and land-use types were key environmental variables affecting the distribution of virus-carrying rodents.

In predictive modeling, temporal features showed the highest contribution to model performance. After excluding temporal features to identify exogenous drivers, land-use types (including forest, cropland, and shrub) showed greater relative importance than climate variables. Climate factors were indirectly associated with HFRS incidence mainly through shaping rodent habitats. The distributions of Apodemus and Rattus responded differently to climate and landscape conditions, and several provinces in eastern China showed high rodent suitability despite moderate reported cases, indicating persistent public health risks.

## Materials and methods

### Ethics statement

This study utilized publicly available case data from the Chinese National Center for Disease Control and Prevention. No individual patient information was used, and no new human participants, human specimens, or animal experiments were involved in this study. Since the data are anonymized and publicly available, ethical approval and consent to participate were not required.

### Data collection

This study collected HFRS case data reported by the Chinese National Center for Disease Control and Prevention (https://www.phsciencedata.cn/Share/index.jsp) from 2005 to 2020, covering 31 provincial-level divisions in mainland China. Data for Hong Kong Special Administrative Region, Macao Special Administrative Region, and Taiwan region were not available in this data. The relevant spatial data included variables divided into three categories:

1. **Biological Features**: Rodent suitability data for HFRS virus carriage based on the MaxEnt model and case data by province, categorized by high-incidence months and provinces.
2. **Environmental Features**: Including climate, ecology, Digital Elevation Model (DEM), and land-use features.
3. **Socioeconomic Features**: Including population data.

The Hantavirus-positive rodent occurrence points used for MaxEnt model construction in this study were derived from two databases:

1) National Earth System Science Data Center, containing approximately 6,000 rodent occurrence records, some of which include Hantavirus detection results. All data in this dataset were independently verified by two individuals to ensure the accuracy of species, locality, and test results. All records with positive Hantavirus test results were selected from this dataset for model construction.
2) NCBI database, from which 1,500 records were obtained. The inclusion criteria were: 1) unambiguous rodent species identification; 2) clear sampling location; 3)explicit pathogen carriage information.

After merging and deduplicating the two data sources, 1630 valid occurrence points were used for MaxEnt model construction. Records lacking virus detection information or with negative test results were not included in this model.

Bioclimatic variables, DEM, Minimum Temperature (Tmin), Maximum Temperature (Tmax), and Precipitation (Prec) data were obtained from WorldClim (https://worldclim.org/) at a resolution of 2.5 arc-minutes. Normalized Difference Vegetation Index (NDVI) and Soil Moisture (SMs) datas were sourced from the National Earth System Science Data Center, National Science & Technology Infrastructure of China (http://www.geodata.cn) with a resolution of 1km. Population data were obtained from the ORNL LandScan Viewer (https://landscan.ornl.gov/) at a resolution of 30 arc-seconds (approximately 1 km). Land use data were sourced from the National Cryosphere Desert Data Center (http://www.ncdc.ac.cn) at a resolution of 30 arc-minutes. All data are available on the aforementioned websites.

### MaxEnt model

The rodent distribution model was constructed using MaxEnt software version 3.4.4. Seventy-five percent of the data were used for training, and 25% were used for testing. Variables were screened using Pearson correlation analysis (r > 0.8), with 8 environmental variables (Bio_6, Bio_5, Bio_3, etc.) ultimately retained [34]. Model optimization was based on the best parameters derived from ENMTools and ENMeval, with 10 runs performed and the average result output in ASCII format [35]. The importance of environmental variables was assessed using Jackknife tests and variable contribution analysis, with AUC values used to judge model accuracy (below 0.5 indicates failure, above 0.9 indicates excellent performance). The prediction results were raster data ranging from 0 to 1, representing suitability probability. Suitability zones were classified using the natural breaks method, and maps were drawn using ArcGIS 10.8.1.

### XGBoost with SHAP model construction methodology

#### 1. Data sources and preprocessing.

The dataset used in this study covers monthly reported cases of hemorrhagic fever with renal syndrome (HFRS) in Chinese provinces from January 2005 to December 2020, along with concurrent meteorological, environmental, and land use data. Meteorological variables include Tmax, Tmin, Prec, SMs, and NDVI. Land use types comprise cropland, forest, grassland, water, barren land, impervious surface, and shrub. The original values for each land use type were pixel proportions, which were subsequently normalized row-wise (per sample) so that each row sums to 1. In addition, the Shannon diversity index of land use was calculated as:


$$\begin{array}{c} land\_diversity=-\sum_{i}p_{i}log(1+p_{i}) \end{array}$$

The habitat suitability variable for hantavirus-carrying rodents was constructed using a MaxEnt model.

#### 2. Feature Engineering.

To incorporate lagged effects and seasonal characteristics of HFRS incidence, the following derived variables were created:

Case lags: Incidence with lags of 1 month (case_lag1) and 12 months (case_lag12).

Annual trend (annual_trend): The rolling mean of incidence over the past 12 months (lagged by 1 period) to represent long-term epidemic levels.

Peak season (peak_season): A binary indicator set to 1 for May-June and November-December, and 0 otherwise, capturing the bimodal seasonal pattern.

Impervious intensity (impervious_intensity): Product of the impervious surface proportion and population density, reflecting the impact of urbanization on transmission..

Temperature range (temp_range): Tmax- Tmin.

All lagged variables were computed separately by province group to preserve the temporal order.

#### 3. Data Partitioning.

Using December 2017 as the cutoff, the data from 2005 to 2020 were split into a training set (2005–2017) and a test set (2018–2020). Within the training set, the first 85% of months (January 2005 to April 2016) were used as the training set and the remaining 15% (May 2016 to December 2017) as the validation set.

#### 4. XGBoost Model Configuration.

An XGBoost regressor was employed with a Tweedie regression objective, which is suitable for positive-skewed data containing many zeros and continuous positive values. Optimal hyperparameters were determined via grid search combined with early-stopping cross-validation. During model training, the root mean square error (RMSE) was monitored on both the training and validation sets, and early stopping was based on validation error. The final model was trained on the combined training and validation sets using the same hyperparameters.

#### 5. Model Evaluation Metrics.

The following metrics were used to evaluate predictive performance on the original incidence scale:

Root Mean Square Error (RMSE): $RMSE=\sqrt{\frac{\text{1}}{n}\sum (y_{i}-{\mathrm{\backslash stackrel}\wedge y}_{i}{)}^{\text{2}}}$
Mean Absolute Error (MAE): $MAE=\frac{\text{1}}{n}\sum \left|y_{i}-\mathrm{\backslash stackrel}\wedge y_{i}\right|$
Coefficient of determination: $R^{\text{2}}=1-\frac{\sum {\text{(y}}_{\text{i}}-{\mathrm{\backslash stackrel}\wedge y}_{i}{)}^{\text{2}}}{\sum (y_{i}-\overset{―}{y}{)}^{\text{2}}}$
Symmetric Mean Absolute Percentage Error (SMAPE): $SMAPE=\frac{200\%}{\text{n}}\sum \frac{\left|y_{i}-{\mathrm{\backslash stackrel}\wedge y}_{i}\right|}{\left|y_{i}\right|+\left|\mathrm{\backslash stackrel}\wedge y_{i}\right|+\varepsilon }$, with $\varepsilon =10^{-8}$

#### 6. Model Interpretation.

To enhance model interpretability, the SHAP (SHapley Additive exPlanations) framework was introduced. Based on the trained XGBoost model, SHAP values were computed for each feature and each sample, quantifying the marginal contribution (direction and magnitude) of each feature to the prediction.

In addition, to specifically assess the nonlinear effects of lagged variables, both GAMs and PDPs (Partial Dependence Plots) were used as complementary analyses. GAMs were fitted with univariate smooth terms to test the significance of nonlinearity (*p*-values). PDPs, based on the XGBoost model, illustrated the average predicted response under marginal changes of a target feature, providing a visual assessment of the marginal effect.

## Supporting information

S1 Table Distribution of the top 20 rodent genera recorded in the literature and total rodent counts by province. (XLSX)

S2 Table AUC values for training and validation sets of 10 Maxent model runs for rodents carrying HVs after variable selection using EMNtools, contribution of each variable, and model averages. (XLSX)

S3 Table Performance evaluation of the constructed XGBoost prediction model. (XLSX)

S4 Table Performance evaluation of the removing temporal features XGBoost model. (XLSX)

S1 Fig ROC curves of the mean Maxent model for 10 rodent species carrying HVs. (TIF)

S2 Fig Mean Jackknife assessment of variable importance for training sets of models for rodents carrying HVs. (TIF)

S3 Fig Mean Jackknife assessment of model AUC for rodents carrying HVs with variables removed and only one variable included. (TIF)

S4 Fig Top 10 SHAP values for the XGBoost prediction model. (TIF)
