# Supplementary material for: Spatial-temporal distribution characteristics of haemorrhagic fever with renal syndrome (HFRS) in 31 provinces and municipalities of P.R. China and multi-factorial prediction of incidence
Source: PLoS Negl Trop Dis. 2026 Sep 11;20(9):e0014619. doi: 10.1371/journal.pntd.0014619 (PMC13585333; doi:10.1371/journal.pntd.0014619)
Supplement: Supplementary file 1 [file pntd.0014619.s010.docx]

Dear Editor and Reviewers,

We greatly appreciate the constructive comments and valuable suggestions from the editor and reviewers.Thank you for giving us the opportunity to revise our manuscript entitled “**Spatial-temporal distribution characteristics of haemorrhagic fever with renal syndrome (HFRS) in 31 provinces and municipalities of P.R.China and multi-factorial prediction of incidence**” (Manuscript ID: PNTD-D-25-01574). We sincerely appreciate your time and the constructive comments provided by the reviewers, which have been invaluable in improving the clarity, methodological rigor, and overall quality of our paper.

We have carefully addressed every point raised in the review. The main revisions include:

## 1. Temporal alignment of rodent and case data

**Response:** We have added a clear explanation in the Materials and Methods section. In addition, we further included 1,500 additional records of hantavirus-positive rodents from the NCBI database to substantially expand the host dataset, and revised the description of the results accordingly. These improvements effectively reduced the limitations caused by insufficient rodent data for ecological suitability prediction and enhanced the reliability of our analysis. (**Page 7, Line 135-147; Page 14, Line 290-292; Page 15, Line 300-309**)

2. Supplementary information on data acquisition , figure size and resolution

**Response:** We have supplemented detailed descriptions for all datasets in the Materials and Methods section. Specifically, we added the data source, spatial resolution, land use classification system, and public access URL for land-use data. We also provided the source, spatial resolution, and access link for the 19 bioclimatic variables, historical precipitation, maximum temperature, and minimum temperature data. In addition, we supplemented the source, spatial resolution, and public access website for population data. (**Page 15, Line 310-320** )

3. Add missing units and labels in figures

**Response:** We have clearly specified the size and resolution of each figure in the corresponding figure captions and added the official direct link to the map base layers (national and regional boundary shapefiles) in the legends of Figure 1, Figure 2, and Figure 5, and ensured that the link is clearly included in the figure captions. Meanwhile, we have supplemented the official approval number and public access identifier of the base maps in the legends of Figure 1C, Figure 2A, Figure 2B, and Figure 5B to comply with copyright requirements and journal guidelines.(**Page 20-21, Line 404-408; Page 22, Line 421-426; Page 25, Line 442-447**)

4. Model weighting logic for high-incidence provinces and clear interpretation of MaxEnt

**Response:** The initial model design originally considered sample weighting for high HFRS incidence provinces and the construction of exogenous interaction variables. After incorporating the newly supplemented and expanded dataset, we reconstructed the XGBoost model. In the revised model, no additional sample weighting was applied to high-incidence provinces, and no exogenous variable interaction terms were generated. We have now described the complete construction procedure of the revised model in detail in the Materials and Methods section, and presented all corresponding modeling results in the Results section. (**Page 8-10, Line 168-193; Page 16-19, Line 336-394**)

### 5. Include an ethics statement

**Response:** We have added a formal ethics statement indicating that the study used only anonymized public surveillance data; no individual identifiers or human/animal experiments were involved, so ethical approval was not required.(**Page 26-27, Line 470-475**)

### 6. Add an explicit testable hypothesis

**Response:** A clear testable hypothesis has been added in the Abstract and Introduction:We hypothesized that climate conditions, land-use structure, rodent host distribution, and their effects are significantly associated with spatiotemporal variation in HFRS incidence in mainland China. (**Page 5, Line 96-98**)

### 7. Strengthen ecological explanations and public health implications

**Response:** We revised the Discussion to strengthen interpretation of host‑environment relationships and provided more targeted public health implications (provincial surveillance, rodent control timing, high‑risk region identification).(**Page 10-13, Line 198-269**)

### 8. Add Author Summary (150–200 words)

**Response:** The Author Summary has been added between the Abstract and Introduction.(**Page 3, Line 47-53**)

### 9. Data availability

**Response:** We have updated the data availability statement: anonymized provincial‑level data are included as supplementary files and are fully open for reuse.

### 10. Revised Financial Disclosure statement

**Response:** This work was supported by the Social Development and Scientific & Technological Research Project of Jiangsu Province (Grant No. JK2025005), the Yiqi Incubation Project of Jiangsu Provincial Center for Disease Control and Prevention (Grant No. 2025YQFH01), the China Postdoctoral Science Foundation General Program (Grant No. 2024M754282), the Key Project of Jiangsu Provincial Administration of Disease Prevention and Control (Grant No. JSJK2026Z00005), the Scientific Research Project of Jiangsu Provincial Health Commission (Grant No. H2023063), and the Scientific Research Project of Hainan Provincial Health Commission (Grant No. WSJK2025MS155), all awarded to W.T. The funders had no role in study design, data collection and analysis, decision to publish, or preparation of the manuscript.(**Page 26, Line 458-468**)

## 11. Other Revisions

Deleted all references to interaction terms that were not included in the model. Deleted references to 3‑month average precipitation (not used in exogenous models). Unified descriptions across Abstract, Results, Discussion, and Conclusion. Improved English writing and flow throughout the manuscript.

We sincerely hope the manuscript now meets the requirements for publication in PLOS Neglected Tropical Diseases.

Thank you again for your time and valuable comments.

Sincerely,Corresponding authorWeilong Tan
