# Supplementary material for: Spatial-temporal distribution characteristics of haemorrhagic fever with renal syndrome (HFRS) in 31 provinces and municipalities of P.R. China and multi-factorial prediction of incidence
Source: PLoS Negl Trop Dis. 2026 Sep 11;20(9):e0014619. doi: 10.1371/journal.pntd.0014619 (PMC13585333; doi:10.1371/journal.pntd.0014619)
Supplement: Peer review history

# Peer review: Decision Letter 0

**DOI:** 10.1371/journal.pntd.0014619.r001

**Authors:** Elvina Viennet

18 Feb 2026

Spatial-temporal distribution characteristics of haemorrhagic fever with renal syndrome (HFRS) in 31 provinces and municipalities of P.R.China and multi-factorial prediction of incidence

PLOS Neglected Tropical Diseases

Dear Dr. Tan,

Thank you for submitting your manuscript to PLOS Neglected Tropical Diseases. After careful consideration, we feel that it has merit but does not fully meet PLOS Neglected Tropical Diseases's publication criteria as it currently stands. Therefore, we invite you to submit a revised version of the manuscript that addresses the points raised during the review process.

Please submit your revised manuscript within by Apr 19 2026 11:59PM. If you will need more time than this to complete your revisions, please reply to this message or contact the journal office at plosntds@plos.org. When you're ready to submit your revision, log on to https://www.editorialmanager.com/pntd/ and select the 'Submissions Needing Revision' folder to locate your manuscript file.

Please include the following items when submitting your revised manuscript:

* A letter that responds to each point raised by the editor and reviewer(s). You should upload this letter as a separate file labeled 'Response to Reviewers'. This file does not need to include responses to any formatting updates and technical items listed in the 'Journal Requirements' section below.

* A marked-up copy of your manuscript that highlights changes made to the original version. You should upload this as a separate file labeled 'Revised Manuscript with Track Changes'.

* An unmarked version of your revised paper without tracked changes. You should upload this as a separate file labeled 'Manuscript'.

If you would like to make changes to your financial disclosure, competing interests statement, or data availability statement, please make these updates within the submission form at the time of resubmission. Guidelines for resubmitting your figure files are available below the reviewer comments at the end of this letter.

We look forward to receiving your revised manuscript.

Kind regards,

Elvina Viennet, PhD

Section Editor

PLOS Neglected Tropical Diseases

Elvina Viennet

Section Editor

PLOS Neglected Tropical Diseases

Shaden Kamhawi

co-Editor-in-Chief

PLOS Neglected Tropical Diseases

orcid.org/0000-0003-4304-636XX

Paul Brindley

co-Editor-in-Chief

PLOS Neglected Tropical Diseases

orcid.org/0000-0003-1765-0002

**Additional Editor Comments:**

This manuscript provides a valuable national analysis of HFRS using multisource environmental and rodent distribution data, but several methodological clarifications are required before acceptance, including temporal alignment of rodent and case data, land use data specifications, model weighting logic, and clearer interpretation of MaxEnt and interaction effects. The authors must also add missing units and labels in figures, include an ethics statement, and articulate an explicit testable hypothesis aligned with their objectives. Recommended (but not required) improvements include strengthening ecological explanations, refining public health implications, improving figure clarity, and enhancing comparisons with existing literature.

**Journal Requirements:**

If the reviewer comments include a recommendation to cite specific previously published works, please review and evaluate these publications to determine whether they are relevant and should be cited. There is no requirement to cite these works unless the editor has indicated otherwise.

1) Please ensure that the CRediT author contributions listed for every co-author are completed accurately and in full.

At this stage, the following Authors/Authors require contributions: Wei-long Tan. Please ensure that the full contributions of each author are acknowledged in the "Add/Edit/Remove Authors" section of our submission form.

The list of CRediT author contributions may be found here: https://journals.plos.org/plosntds/s/authorship#loc-author-contributions

2) Please provide an Author Summary. This should appear in your manuscript between the Abstract (if applicable) and the Introduction, and should be 150-200 words long. The aim should be to make your findings accessible to a wide audience that includes both scientists and non-scientists. Sample summaries can be found on our website under Submission Guidelines:

https://journals.plos.org/plosntds/s/submission-guidelines#loc-parts-of-a-submission

3) Please upload all main figures as separate Figure files in .tif or .eps format. For more information about how to convert and format your figure files please see our guidelines:

https://journals.plos.org/plosntds/s/figures

4) We have noticed that you have uploaded Supporting Information files, but you have not included a list of legends. Please add a full list of legends for your Supporting Information files after the references list.

5) Some material included in your submission may be copyrighted. According to PLOSu2019s copyright policy, authors who use figures or other material (e.g., graphics, clipart, maps) from another author or copyright holder must demonstrate or obtain permission to publish this material under the Creative Commons Attribution 4.0 International (CC BY 4.0) License used by PLOS journals. Please closely review the details of PLOSu2019s copyright requirements here: PLOS Licenses and Copyright. If you need to request permissions from a copyright holder, you may use PLOS's Copyright Content Permission form.

Please respond directly to this email and provide any known details concerning your material's license terms and permissions required for reuse, even if you have not yet obtained copyright permissions or are unsure of your material's copyright compatibility. Once you have responded and addressed all other outstanding technical requirements, you may resubmit your manuscript within Editorial Manager.

Potential Copyright Issues:

i) Figures 1C, 2A-2C, 5B, S6, S9, S12, S13, S14, S15, and S16. Please (a) provide a direct link to the base layer of the map (i.e., the country or region border shape) and ensure this is also included in the figure legend; and (b) provide a link to the terms of use / license information for the base layer image or shapefile. We cannot publish proprietary or copyrighted maps (e.g. Google Maps, Mapquest) and the terms of use for your map base layer must be compatible with our CC BY 4.0 license.

Note: if you created the map in a software program like R or ArcGIS, please locate and indicate the source of the basemap shapefile onto which data has been plotted.

If your map was obtained from a copyrighted source please amend the figure so that the base map used is from an openly available source. Alternatively, please provide explicit written permission from the copyright holder granting you the right to publish the material under our CC BY 4.0 license.

If you are unsure whether you can use a map or not, please do reach out and we will be able to help you. The following websites are good examples of where you can source open access or public domain maps:

* U.S. Geological Survey (USGS) - All maps are in the public domain. (http://www.usgs.gov)

* PlaniGlobe - All maps are published under a Creative Commons license so please cite u201cPlaniGlobe, http://www.planiglobe.com, CC BY 2.0u201d in the image credit after the caption. (http://www.planiglobe.com/?lang=enl)

* Natural Earth - All maps are public domain. (http://www.naturalearthdata.com/about/terms-of-use/).

6) In the online submission form, you indicated that The source data files were available from njcdc@163.com upon reasonable request.. All PLOS journals now require all data underlying the findings described in their manuscript to be freely available to other researchers, either

1. In a public repository

2. Within the manuscript itself

3. Uploaded as supplementary information.

This policy applies to all data except where public deposition would breach compliance with the protocol approved by your research ethics board. If your data cannot be made publicly available for ethical or legal reasons (e.g., public availability would compromise patient privacy), please explain your reasons by return email and your exemption request will be escalated to the editor for approval. Your exemption request will be handled independently and will not hold up the peer review process, but will need to be resolved should your manuscript be accepted for publication. One of the Editorial team will then be in touch if there are any issues.

7) Please amend your detailed Financial Disclosure statement. This is published with the article. It must therefore be completed in full sentences and contain the exact wording you wish to be published.

1) State the initials, alongside each funding source, of each author to receive each grant. For example: "This work was supported by the National Institutes of Health (####### to AM; ###### to CJ) and the National Science Foundation (###### to AM)."

2) State what role the funders took in the study. If the funders had no role in your study, please state: "The funders had no role in study design, data collection and analysis, decision to publish, or preparation of the manuscript."

3) If any authors received a salary from any of your funders, please state which authors and which funders..

If you did not receive any funding for this study, please simply state: u201cThe authors received no specific funding for this work.u201d

8) Your current Financial Disclosure states, "Yes ↳ Please add funding details. social development and scientific & technological research project (Grant numbers BK20221196, JK2023005 and JK2023006) ↳ Please select the country of your main research funder (please select carefully as in some cases this is used in fee calculation). CHINA - CN".

However, your funding information on the submission form indicates different grant numbers 2025YQFH01, BK20221196, BE2022682.

Please indicate by return email the full and correct funding information for your study and confirm the order in which funding contributions should appear. Please be sure to indicate whether the funders played any role in the study design, data collection and analysis, decision to publish, or preparation of the manuscript.

**Reviewers' Comments:**

Reviewer's Responses to Questions

**Key Review Criteria Required for Acceptance?**

As you describe the new analyses required for acceptance, please consider the following:

**Methods**

-Are the objectives of the study clearly articulated with a clear testable hypothesis stated?

-Is the study design appropriate to address the stated objectives?

-Is the population clearly described and appropriate for the hypothesis being tested?

-Is the sample size sufficient to ensure adequate power to address the hypothesis being tested?

-Were correct statistical analysis used to support conclusions?

-Are there concerns about ethical or regulatory requirements being met?

Reviewer #1: Yes

Reviewer #2: 1. Are the objectives of the study clearly articulated with a clear testable hypothesis stated?

Answer: Partially

The objective is clearly stated: to analyze spatiotemporal distribution of HFRS and predict incidence using multi-source data. The limitation is clear, formal testable hypothesis is not explicitly stated. For example, there is no phrasing that mention any hypothesis on climate, host, and land-use features significantly influence HFRS incidence at the provincial scale.”

2. Is the study design appropriate to address the stated objectives?

Yes.

A retrospective, observational, nationwide analysis using case records, environmental/climate data, and rodent distributions is suitable.

Integration of XGBoost-SHAP, MaxEnt, and GAM models is appropriate for modelling complex multi-factorial influences.

3. Is the population clearly described and appropriate for the hypothesis being tested?

Mostly Yes.

Population: 182,865 HFRS cases from 31 provinces in China (2005–2020).

Age distributions, geographic hotspots, and temporal trends are described.

Some rodent host populations have sparse data (140 virus-positive records), which may limit ecological inference.

4. Is the sample size sufficient to ensure adequate power to address the hypothesis being tested?

Yes. 182,865 cases. Rodent host data: Possibly underpowered. Only 140 positive records from over 6,000 rodents.

5. Were correct statistical analysis used to support conclusions?

Mostly yes. The limitation SHAP contributions and GAM effect sizes are sometimes ambiguous or not fully quantified.

High R² (~0.967) suggests possible overfitting, especially since temporal features dominate.

6. Are there concerns about ethical or regulatory requirements being met?

Likely no major concerns. Study uses retrospective human case data (likely de-identified) and rodent surveillance data. Manuscript does not explicitly mention IRB approval or data permission. Recommendation: Include statement that ethical guidelines and relevant approvals were followed.

**********

**Results**

-Does the analysis presented match the analysis plan?

-Are the results clearly and completely presented?

-Are the figures (Tables, Images) of sufficient quality for clarity?

Reviewer #1: Yes

Reviewer #2: 1. Does the analysis presented match the analysis plan? Yes

Some parts of the results are vague and not fully quantified. Interaction terms - Rodent- forest-edge, Forest-Shrub-Rodent are mentioned but not clearly explained.

2. Are the results clearly and completely presented?

Temporal trends, seasonal peaks, and age distribution are clearly described. Key predictive variables (temporal lags, land-use, climate) are identified. Geographic distribution of cases and rodent species is presented with relevant highlights. Quantitative details and visual representation need improvement.

3. Are the figures (Tables, Images) of sufficient quality for clarity?

Yes.

**********

**Conclusions**

-Are the conclusions supported by the data presented?

-Are the limitations of analysis clearly described?

-Do the authors discuss how these data can be helpful to advance our understanding of the topic under study?

-Is public health relevance addressed?

Reviewer #1: Yes

Reviewer #2: 1. Are the conclusions supported by the data presented?

The conclusions summarize the key findings: land-use features (Forest, Shrub, Cropland) and climate indices (3-month average precipitation and temperature) influence HFRS incidence.

2, Are the limitations of analysis clearly described?

Some limitations are acknowledged in the Discussion: underreporting, provincial-level analysis, sparse rodent data, interactions not fully explored.

3. Do the authors discuss how these data can be helpful to advance our understanding of the topic under study?

Yes. But could be more explicit about scientific contribution: e.g., advancing predictive epidemiology, understanding host-mediated climate effects, or multi-factor interaction modelling.

Is public health relevance addressed?

Conclusion mentions providing key scientific evidence for policymakers to formulate targeted prevention and control strategies and elaborates on rodent control, early warning, urbanization, and seasonal intervention strategies. The conclusion could link findings more directly to specific interventions.

**********

**Editorial and Data Presentation Modifications?**

Use this section for editorial suggestions as well as relatively minor modifications of existing data that would enhance clarity. If the only modifications needed are minor and/or editorial, you may wish to recommend “Minor Revision” or “Accept”.

Reviewer #1: Minor Revision

Reviewer #2: The manuscript is scientifically strong, but clarity and readability can be improved with editorial and presentation changes:

1. Use active voice consistently.

Break long paragraphs for readability.

Clearly state the testable hypothesis.

Streamline repetitive statements and overconfident phrasing.

Include an ethical approval statement.

2. Data Presentation:

Ensure all figures and tables have clear legends, units, scales, and labels.

Visualize key interactions

Move key supplemental figures into the main text if they show primary findings.

3. Minor Adjustments:

Correct typos and inconsistent capitalization

Define all abbreviations.

Consider a flow diagram showing the analysis workflow.

Recommendation: These are mostly minor editorial and presentation improvements. If addressed, the manuscript would be suitable for Minor Revision.

**********

**Summary and General Comments**

Use this section to provide overall comments, discuss strengths/weaknesses of the study, novelty, significance, general execution and scholarship. You may also include additional comments for the author, including concerns about dual publication, research ethics, or publication ethics. If requesting major revision, please articulate the new experiments that are needed.

Reviewer #1: In China, HFRS is a highly prevalent and severe rodent-borne disease. Although the overall incidence of HFRS nationwide has shown a steady downward trend in recent years, the epidemic situation remains non-negligible. Outdoor occupational groups such as farmers face persistently high infection risks due to frequent exposure to rodent habitats. Meanwhile, China's extensive geographical span, diverse climate types, rapid urbanization, and land-use changes are constantly altering the reproductive cycles and habitat distributions of rodent hosts, further exacerbating the complexity of virus transmission and posing tremendous challenges to precise prevention and control. This study focused on Hemorrhagic Fever with Renal Syndrome (HFRS) across 31 provinces in China from 2005 to 2020. It integrates multi-source spatiotemporal data. Meanwhile, it combines the MaxEnt model to analyze rodent distribution and key environmental variables, systematically exploring the influencing factors of HFRS incidence.

Reviewer #2: This manuscript presents a comprehensive nationwide study of Hemorrhagic Fever with Renal Syndrome (HFRS) in China, analyzing multi-source spatiotemporal data from 2005 to 2020. The authors aim to systematically assess the determinants of HFRS incidence including climate, land-use, host, and socioeconomic factors and to construct a predictive model using XGBoost-SHAP integrated with MaxEnt ecological niche modeling and GAM temporal analysis.

This manuscript is highly significant, methodologically innovative, and has strong public health relevance. The study provides valuable insights into HFRS spatiotemporal trends, host-environment interactions, and predictive modeling.

However, major revisions are needed to improve clarity, quantification, interpretation, and presentation before the manuscript can be accepted.

Once addressed, it has strong potential for publication and will make an important contribution to HFRS epidemiology and predictive modeling in infectious diseases.

**********

PLOS authors have the option to publish the peer review history of their article (what does this mean?). If published, this will include your full peer review and any attached files.

If you choose “no”, your identity will remain anonymous but your review may still be made public.

**Do you want your identity to be public for this peer review?**  For information about this choice, including consent withdrawal, please see our Privacy Policy.

Reviewer #1: No

Reviewer #2: **Yes:** Adama Ahmad Abubakar

**Figure resubmission:**

While revising your submission, we strongly recommend that you use PLOS’s NAAS tool (https://ngplosjournals.pagemajik.ai/artanalysis) to test your figure files. NAAS can convert your figure files to the TIFF file type and meet basic requirements (such as print size, resolution), or provide you with a report on issues that do not meet our requirements and that NAAS cannot fix.

**Reproducibility:**

To enhance the reproducibility of your results, we recommend that authors of applicable studies deposit laboratory protocols in protocols.io, where a protocol can be assigned its own identifier (DOI) such that it can be cited independently in the future. Additionally, PLOS ONE offers an option to publish peer-reviewed clinical study protocols. Read more information on sharing protocols at https://plos.org/protocols?utm_medium=editorial-email&utm_source=authorletters&utm_campaign=protocols

# Author comment: Author response to Decision Letter 1

**DOI:** 10.1371/journal.pntd.0014619.r002

13 May 2026

Attachment Submitted filename: Response to Reviewers(1).docx

# Peer review: Decision Letter 1

**DOI:** 10.1371/journal.pntd.0014619.r003

**Authors:** Elvina Viennet

27 Jul 2026

Dear Mr Tan,

We are pleased to inform you that your manuscript 'Spatial-temporal distribution characteristics of haemorrhagic fever with renal syndrome (HFRS) in 31 provinces and municipalities of P.R.China and multi-factorial prediction of incidence' has been provisionally accepted for publication in PLOS Neglected Tropical Diseases.

Before your manuscript can be formally accepted you will need to complete some formatting changes, which you will receive in a follow up email. A member of our team will be in touch with a set of requests.

Please note that your manuscript will not be scheduled for publication until you have made the required changes, so a swift response is appreciated.

IMPORTANT: The editorial review process is now complete. PLOS will only permit corrections to spelling, formatting or significant scientific errors from this point onwards. Requests for major changes, or any which affect the scientific understanding of your work, will cause delays to the publication date of your manuscript.

Should you, your institution's press office or the journal office choose to press release your paper, you will automatically be opted out of early publication. We ask that you notify us now if you or your institution is planning to press release the article. All press must be co-ordinated with PLOS.

Thank you again for supporting Open Access publishing; we are looking forward to publishing your work in PLOS Neglected Tropical Diseases.

Best regards,

Elvina Viennet, PhD

Section Editor

PLOS Neglected Tropical Diseases

Elvina Viennet

Section Editor

PLOS Neglected Tropical Diseases

Shaden Kamhawi

co-Editor-in-Chief

PLOS Neglected Tropical Diseases

orcid.org/0000-0003-4304-636XX

Paul Brindley

co-Editor-in-Chief

PLOS Neglected Tropical Diseases

orcid.org/0000-0003-1765-0002

***********************************************************

Reviewer's Responses to Questions

**Key Review Criteria Required for Acceptance?**

As you describe the new analyses required for acceptance, please consider the following:

**Methods**

-Are the objectives of the study clearly articulated with a clear testable hypothesis stated?

-Is the study design appropriate to address the stated objectives?

-Is the population clearly described and appropriate for the hypothesis being tested?

-Is the sample size sufficient to ensure adequate power to address the hypothesis being tested?

-Were correct statistical analysis used to support conclusions?

-Are there concerns about ethical or regulatory requirements being met?

Reviewer #1: Yes

Reviewer #2: -Are the objectives of the study clearly articulated with a clear testable hypothesis stated? - YES

-Is the study design appropriate to address the stated objectives? - YES

-Is the population clearly described and appropriate for the hypothesis being tested? - YES

-Is the sample size sufficient to ensure adequate power to address the hypothesis being tested? - YES (Comment: The conclusions are generally supported by these analyses. However, as acknowledged by the authors, causal inference is limited because this is an observational ecological study.

-Were correct statistical analysis used to support conclusions? - YES (Minor limitation)

-Are there concerns about ethical or regulatory requirements being met? No major concern

**********

**Results**

-Does the analysis presented match the analysis plan?

-Are the results clearly and completely presented?

-Are the figures (Tables, Images) of sufficient quality for clarity?

Reviewer #1: Yes

Reviewer #2: -Does the analysis presented match the analysis plan? YES

-Are the results clearly and completely presented? - YES

-Are the figures (Tables, Images) of sufficient quality for clarity? - YES

**********

**Conclusions**

-Are the conclusions supported by the data presented?

-Are the limitations of analysis clearly described?

-Do the authors discuss how these data can be helpful to advance our understanding of the topic under study?

-Is public health relevance addressed?

Reviewer #1: Yes

Reviewer #2: -Are the conclusions supported by the data presented? - YES

-Are the limitations of analysis clearly described? - YES

-Do the authors discuss how these data can be helpful to advance our understanding of the topic under study? -YES

-Is public health relevance addressed? - YES

**********

**Editorial and Data Presentation Modifications?**

Use this section for editorial suggestions as well as relatively minor modifications of existing data that would enhance clarity. If the only modifications needed are minor and/or editorial, you may wish to recommend “Minor Revision” or “Accept”.

Reviewer #1: Accept

Reviewer #2: The following should be critically looked into:

1. Language and grammar: Although the manuscript has been edited, there are still occasional grammatical errors, awkward sentence constructions, and typographical mistakes e.g. distributiob, occurance, ridk, Eraly.

E.g.2. Consistency in terminology to ensure consistent use of terminology throughout the manuscript. example, HFRS incidence, incidence cases, and HFRS cases are used interchangeably. Similarly, maintain consistent capitalization and formatting for terms such as MaxEnt, XGBoost, and SHAP.

3. Abbreviations should be defined at first mention and used consistently throughout the text, figures, and supplementary materials. Some environmental variables (e.g., Bio_5, Bio_6, Bio_13) are explained only in figure legends rather than within the main text.

**********

**Summary and General Comments**

Use this section to provide overall comments, discuss strengths/weaknesses of the study, novelty, significance, general execution and scholarship. You may also include additional comments for the author, including concerns about dual publication, research ethics, or publication ethics. If requesting major revision, please articulate the new experiments that are needed.

Reviewer #1: I have no further comments and recommend acceptance for publication.

Reviewer #2: This manuscript presents a nationwide ecological analysis of the spatial-temporal distribution of haemorrhagic fever with renal syndrome (HFRS) in mainland China from 2005 to 2020. By integrating surveillance, climatic, land-use, socioeconomic, and rodent distribution data, the authors develop an XGBoost-SHAP prediction model complemented by MaxEnt ecological niche modelling to identify key determinants of HFRS incidence.

The study addresses an important public health problem and contributes to the growing application of machine learning approaches in infectious disease surveillance and prediction.

The major strengths of the study include the use of a large national dataset spanning 16 years, integration of multiple environmental and ecological data sources, application of appropriate machine learning and spatial analytical methods, and incorporation of SHAP values to improve model interpretability. The manuscript is generally well structured, and the analyses are consistent with the stated objectives. The findings provide useful insights into the relative contributions of temporal trends, land-use characteristics, climate, and rodent habitats to HFRS transmission, with potential implications for disease surveillance and early warning systems.

The revised manuscript has addressed many of the concerns raised during peer review, including the inclusion of a clear, testable hypothesis, expanded description of data sources, clarification of model construction, addition of an ethics statement, and improved discussion of public health implications. These revisions have substantially strengthened the scientific quality and transparency of the manuscript.

Nevertheless, several minor issues remain. The manuscript would benefit from a final professional English language edit to correct grammatical errors, typographical mistakes, and improve sentence flow. Statistical reporting could also be strengthened by including confidence intervals and effect size estimates where appropriate, rather than relying primarily on model importance measures. Although the authors appropriately acknowledge the limitations of ecological study design, incomplete rodent surveillance data, potential underreporting of HFRS cases, and the lack of finer spatial resolution, it would also be helpful to discuss the absence of external validation of the prediction model, which may affect its generalizability. Additionally, a summary table presenting the most influential predictors and their relative contributions would improve the accessibility of the findings.

No major concerns regarding research ethics or publication ethics were identified. The study uses anonymized, publicly available surveillance data, provides an appropriate ethics statement, declares funding sources and competing interests, and includes a data availability statement consistent with journal requirements.

Overall, this is a scientifically sound and relevant study that makes a meaningful contribution to understanding the ecological drivers of HFRS and demonstrates the value of integrating ecological niche modelling with interpretable machine learning for infectious disease prediction. The remaining issues are primarily editorial and relate to clarity of presentation rather than scientific validity. Therefore, I recommend Minor Revision before publication.

**********

PLOS authors have the option to publish the peer review history of their article (what does this mean?). If published, this will include your full peer review and any attached files.

If you choose “no”, your identity will remain anonymous but your review may still be made public.

**Do you want your identity to be public for this peer review?**  For information about this choice, including consent withdrawal, please see our Privacy Policy.

Reviewer #1: No

Reviewer #2: **Yes:** Mrs. Adama Ahmad Abubakar

# Editor report: Acceptance letter

**DOI:** 10.1371/journal.pntd.0014619.r004

**Authors:** Elvina Viennet

Dear Mr Tan,

We are delighted to inform you that your manuscript, "Spatial-temporal distribution characteristics of haemorrhagic fever with renal syndrome (HFRS) in 31 provinces and municipalities of P.R.China and multi-factorial prediction of incidence," has been formally accepted for publication in PLOS Neglected Tropical Diseases.

We have now passed your article onto the PLOS Production Department who will complete the rest of the publication process. All authors will receive a confirmation email upon publication.

The corresponding author will soon be receiving a typeset proof for review, to ensure errors have not been introduced during production. Please review the PDF proof of your manuscript carefully, as this is the last chance to correct any scientific or type-setting errors. Please note that major changes, or those which affect the scientific understanding of the work, will likely cause delays to the publication date of your manuscript. Note: Proofs for Front Matter articles (Editorial, Viewpoint, Symposium, Review, etc...) are generated on a different schedule and may not be made available as quickly.

Soon after your final files are uploaded, the early version of your manuscript will be published online unless you opted out of this process. The date of the early version will be your article's publication date. The final article will be published to the same URL, and all versions of the paper will be accessible to readers.

For Research Articles, you will receive an invoice from PLOS for your publication fee after your manuscript has reached the completed accept phase. If you receive an email requesting payment before acceptance or for any other service, this may be a phishing scheme. Learn how to identify phishing emails and protect your accounts at https://explore.plos.org/phishing.

Thank you again for supporting open-access publishing; we are looking forward to publishing your work in PLOS Neglected Tropical Diseases.

Best regards,

Shaden Kamhawi

co-Editor-in-Chief

PLOS Neglected Tropical Diseases

Paul Brindley

co-Editor-in-Chief

PLOS Neglected Tropical Diseases
